# Supplementary material for: Structure-Based Analysis of Protein Cluster Size for Super-Resolution Microscopy in the Nervous System
Source: Biomedicines. 2022 Jan 27;10(2):295. doi: 10.3390/biomedicines10020295 (PMC8869149; doi:10.3390/biomedicines10020295)
Supplement: Supplementary file 1 [file biomedicines-10-00295-s001.zip › biomedicines-1564258-supplementary.pdf]

Supplementary Note 1: Concepts of SBA and derivation of algorithm

Localization-based super-resolution techniques enable fluorescent microscopy to resolve nanoscale structure. In these imaging techniques, localization analysis records the position of fluorophores as they switch between light and dark states. During these process, the detected blinking spots were taken as the PSF of emitting point sources and the central coordinates of the PSFs were registered as the localization position; ideally, dispersed and isolated fluorophores afford maximum localization accuracy and precision. However, in biological application, most target structures exist as protein clusters having physical sizes. This issue makes local size measurement by localization-based super-resolution microscopy difficult because for a spatially correlated distribution, the PSFs generated by neighboring emitting fluorophores can overlap spatially. In addition, the process of localization records only the central position of each fluorophore, and information registered in the lateral width of the PSF is discarded during fitting. As a result, in STORM or PALM images, parameters such as the FWHM are still used as an indefinite estimate of the resolved structure size. We designed Structure-Based Analysis (SBA), which is a structure-based method utilizing the structural information of the desired target by incorporating the structural function corresponding to the underlying protein structure of the target localization cluster. In the following we discuss the derivation of SBA.

In signal processing, light diffraction is equivalent to the process of convolution, in which the structural information of the emitting source is incorporated into the PSF of the imaging apparatus. Mathematically, the diffraction-limited spatial emitting profile $D(x)$ is the convolution of the PSF and the original emission distribution denoted by the structural function $S(x)$, which reveals the structure of the emitter:

$$D(x)=\{S*PSF\}(x) \qquad \text{eq (1)}$$

Here, x is the position along the principle axis and the convolution in equation 1 can be explicitly written into the integral form, that is

$$D(x)=\int S(x') \, PSF(x-x') \, dx' \qquad \text{eq (2)}$$

Here, the PSF for a point source can generally be described by the Airy function, which can be further approximated by a Gaussian function denoted by $G(x)$. Thus, equation 2 can be further expressed into the integral of the structural function multiplied by the Gaussian PSF, i.e.:

$$D(x)=\int S(x') \, G(x-x') \, dx' = SBA(x) \qquad \text{eq (3)}$$

Equation 3 describes the general form of one-dimensional spatial emission profile generated by a structural function denoted by $S(x)$. We called the function in equation 3 Structure-based Analysis Function abbreviated by $SBA(x)$, which was further used in SBA.

Supplementary Note 2: Numerical derivation of spherical SBSF.

To perform SBA by utilizing the information incorporated in the structural function $S(x)$, explicit derivation of the Structure-based Analysis Function $SBA(x)$ is necessary. In the following we derived the SBA for a spherical emitting source, which may physically represent a spherical protein inclusion or a disc shape ellipsoidal cluster such as the postsynaptic density.

Assuming a spherical or ellipsoidal surface with homogeneous labeled fluorophores, the unit emitted light flux along the surface is constant, and thus, for a certain unit area on the surface, the corresponding light flux detected on the image plane is proportional to the cosine of the dihedral angle between the image plane and the tangent plane of the unit emitting surface area. Mathematically, this intensity emission line profile is expressed as

$$S = S_0 \cos\theta \qquad \text{eq (4)}$$

Where S_0 is the constant of the emission intensity and θ is the dihedral angle between the image plane and the tangent plane the unit surface. To incorporate the structural information of the target structure, in this case, the diameter of the sphere, here θ can further be substituted by two parameters: the position away from the center of the cluster x, and the diameter of the emitting spherical cluster d.

$$\theta = \cos^{-1} \frac{\sqrt{(d\,/\,2)^2 - x^2}}{d\,/\,2} \qquad \text{eq (5)}$$

And equation 4 can be re-written as

$$S = S_0 \frac{\sqrt{(d\,/\,2)^2 - x^2}}{d\,/\,2} \qquad \text{eq (6)}$$

Where d and X represent structural information of the target cluster: the diameter of the sphere and the position of the emitting point relative to the center of the sphere, respectively. The Structure-based Analysis Function $SBA(x)$ of a spherical or ellipsoidal structure is:

$$SBA(x) = \int S_0 \frac{\sqrt{(d/2)^2 - x'^2}}{d/2}\, G(x - x')dx' \qquad \text{eq (7)}$$

Since it may not always be possible to calculate the indefinite integral of a general SBA function represented by equation 7, we use the method of discrete convolution for calculation. The general equation of discrete convolution of two functions, A and B, is

$$\{A * B\}(x) = \sum_k A(k)B(x - k) \qquad \text{eq (8)}$$

Next, we derive the discrete approximation of the structural function. Considering a general structural function $S(x)$, it can be expressed in to the summation of a collection of impulse functions I to obtain the general form of the corresponding discrete structural function:

$$S(x) \sim \sum_{k} S(k) I(x-k) \qquad \text{eq (9)}$$

Next, considering a spherical structure, the spherical structural function in equation 6 is re-written into a summation of impulse functions, and this gives:

$$S_0 \frac{\sqrt{(^{d}/_{2})^2 - x^2}}{d/2} \sim \sum_{k=-n}^{n} S_0 \frac{\sqrt{(d/2)^2 - \left(\frac{dk}{2n}\right)^2}}{d/2} I\left(x - \frac{dk}{2n}\right) \qquad \text{eq (10)}$$

Here, the a single impulse function I in equations 9 and 10 is

$$I\left(x - \frac{dk}{2n}\right) = 1, \frac{d(k-1/2)}{2n} \leq x \leq \frac{d(k+1/2)}{2n}$$

$$I\left(x - \frac{dk}{2n}\right) = 0, \frac{d\left(k - {}^{1}/_{2}\right)}{2n} \geq x \; or \; \frac{d\left(k + {}^{1}/_{2}\right)}{2n} \leq x \qquad \text{eq (11)}$$

The discrete approximation of the spherical structural function is expressed in equation 10 and n is determined by letting the corresponding width of a single impulse be in closest proximity to the achievable resolution of the imaging device; mathematically, n is chosen as the positive integer minimizing the following function:

$$\left(\frac{FWHM}{2n+1} - R\right)^2 \qquad \text{eq (12)}$$

FWHM here represents the full width at half-maximum estimated from the image and R is the pixel size of the STORM image. Substituting the discrete form of the spherical structural function in equation 10 into equation 7, the SBA function becomes:

$$SBA(x) = \sum_{k=-n}^{n} S_0 \frac{\sqrt{\left({}^{d}/_{2}\right)^2 - \left(\frac{dk}{2n}\right)^2}}{d/2} G(x - \frac{dk}{2n}) \qquad \text{eq (13)}$$

Equation 13 is the explicit form of the SBA function for a spherical emitting source, where d is the diameter of the spherical or ellipsoid structure and constitutes the spatial information about the structural dimension of the emitting source. The Gaussian PSF here, can be explicitly expressed as

$$G(x - x_0) = G_0 e^{-C_0 (x - x_0)^2} \qquad \text{eq (14)}$$

By substituting equation 14 into equation 13, the final SBA function for a spherical emitting source derived by discrete convolution is found to be

$$SBA(x) = \sum_{k=-n}^{n} S_0 \frac{\sqrt{\left({}^{d}/_{2}\right)^2 - \left(\frac{dk}{2n}\right)^2}}{d\ /\ 2} G_0 e^{-C_0\left(x-\frac{dk}{2n}\right)^2} \quad \text{eq (15)}$$

Which is a summation with finite terms that can be directly expanded.

Supplementary Figure S1

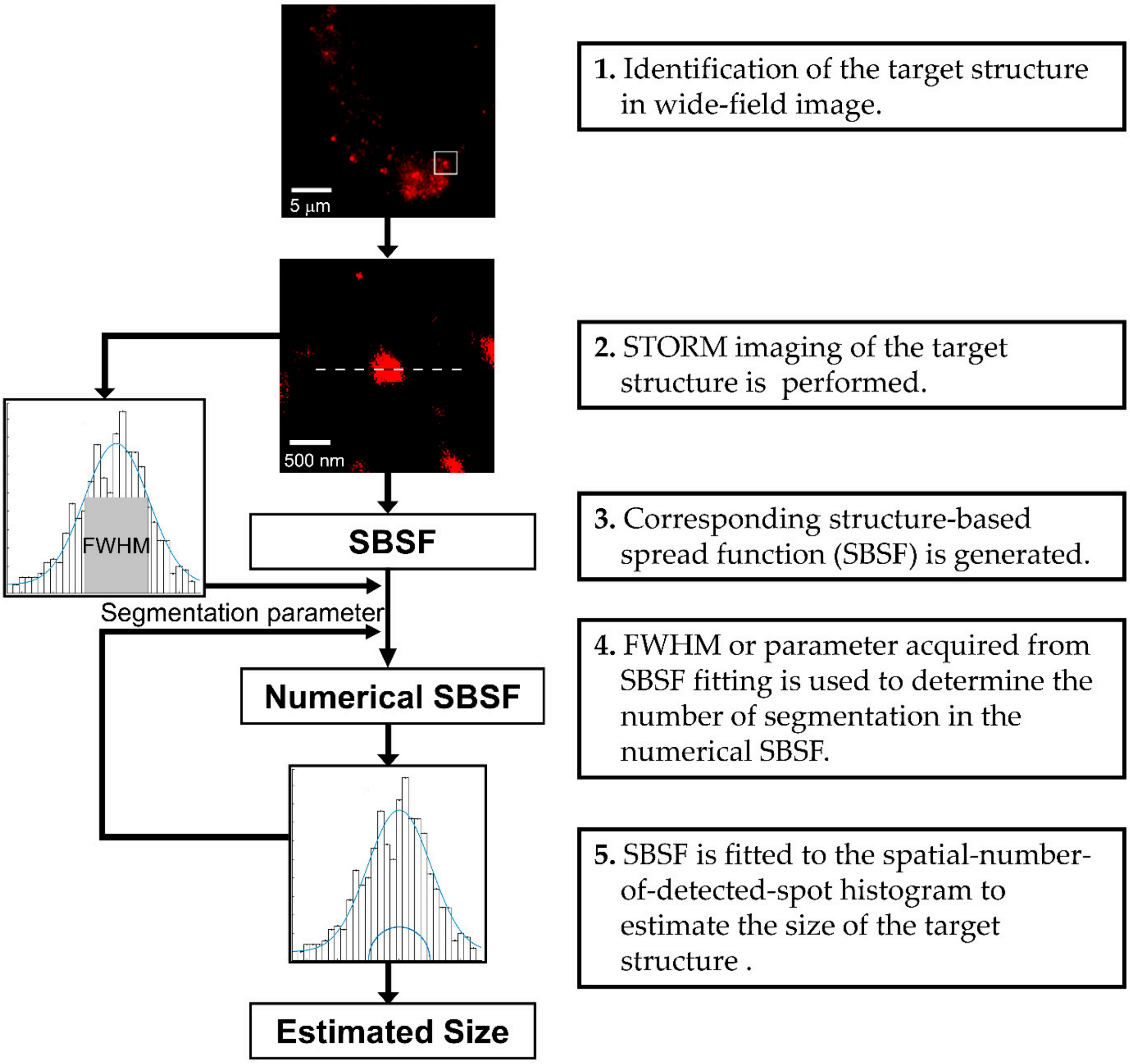

Figure S1. Schematic illustration of structure-based analysis (SBA).
